# Screening for Substance Use Disorders in Primary Care Settings: A Systematic Review

**DOI:** 10.1192/j.eurpsy.2026.10490

**Published:** 2026-07-31

**Authors:** L. Li

**Affiliations:** Psychiatry and Behavioral Neurobiology, University of Alabama at Birmingham, Birmingham, United States

## Abstract

**Introduction:**

Population-based screenings for substance use are highly recommended, but little is known about the screening status for substances in primary care settings (PCSs).

**Objectives:**

This systematic review aims to understand what screening tools are used, barriers that impact screenings, and opportunities to improve screenings.

**Methods:**

Articles focusing on the utilization of screening tools for substance use disorders (SUDs) within PCSs were considered for inclusion. Searches on PubMed and Embase databases were conducted from April 25, 2022 through July 22, 2022, which yielded 487 results; only 32 articles meeting the inclusion and exclusion criteria were included.

**Results:**

Among all substances, alcohol is the most screened for at PCSs, with 20 studies included in this article. This was followed by opioids (n = 11), other illicit drugs (n = 8), cannabis (n = 6), and nicotine (n = 2). The Alcohol Use Disorders Identification Test was the screening tool most used. Most studies utilized different screening tools to screen for the same substances. Identified barriers include no referral to treatment upon positive screenings, lack of training in providing intervention, and no recognition of the effectiveness of intervention services.

**Conclusions:**

Our search resulted in only 32 studies focusing on substance screening at PCSs, indicating that substance screening may not be conducted as recommended or formally. Integrating patient-centered SUDs screening in PCSs is challenging but could be feasible via implementation strategies to address barriers. Future studies focusing on the feasibility, acceptability, and effectiveness of screening tools in PCSs may increase the screening rates for early identification and intervention for SUDs.

**Disclosure of Interest:**

None Declared

